# Silicea

**Published:** 1887-06

**Authors:** A. McNeil

**Affiliations:** San Francisco, Cal.


### SILICEA.

A Lecture by Professor A. McNeil, M. D., San Francisco, Cal.

This remedy is a pure antipsoric. There are such remedies as Arsen., Lycop., and even Sulph., which are adapted to acute as well as chronic diseases. But with Silicea this is not the case. While it has some febrile symptoms, they are those of hectic as distinguished from other fevers.

In this way it is adapted to scrofulous diseases, and it is diseases of this character in their many protean forms that Sil. cures.

In all the cases cured by Silicea, psora is discoverable. The child in whom the osseous development is imperfect, as revealed by the large, sweating head, open fontanelles, slow dentition, crooked legs, and enlarged ulcerated glands and unclean nose of the children of large growth.

We find important mental indications. Solicitude concerning his spiritual welfare, in this resembling Sulph. and Verat. alb. He labors under the delusion that he is in two places at once. He lacks confidence in his own ability, yet when he begins he does well, and is of a yielding disposition, faint-hearted and anxious. There may be a suicidal tendency, wishing to drown himself, and is fearful of pursuing enemies, like the Bell. patient, but it is almost the only thing which the two have in common.

The patient may be haunted with hallucinations of corpses. Another mental condition is where she occupies herself with pins, counts them and hunts for them, such as we see in certain mental conditions resembling imbecility.

The Silicea patient is much troubled with vertigo ; he is dizzy even while asleep, and when sleepy ; sometimes it appears to rise up from the nape of the neck, and with an inclination to fall forward, and while looking up, like the Pulsatilla patient, and, the strangest of all, is dizzy only when lying on the left side.

In scrofulous women particularly we will find this an indispensable remedy in headaches when distinguished by the following characteristics:

It rises from the nape of the neck to the vertex, and thence over the eyes (with Sanguinaria it is the same, but settles over the right eye; with Gelsimium it is much the same, but in a different class of patients).

There is another Silicea headache with nausea, and is aggravated by noise and motion, and even by the jar of the bed, all reminding us of the Bell. headache, but it lacks the red face and heat of the latter. It is relieved by enveloping the head in woolen cloths or in hot compresses, like Magnesia mur., but the latter is relieved in the open air, while Silicea is aggravated thereby ; you must remember that it is the heat not the pressure that relieves the Silicea headache.

There is also a Silicea headache which is a sharp, darting pain from the occiput to the eyeballs, especially to the right one, with a steady ache of the eyeballs, which are sore and tender to touch.

There are also cases with painful jerks in the forehead, and are renewed and aggravated by turning quickly, stooping, and talking ; some time after the headache the patient sees spectres ; with Psorin he sees them before. The Silicea headache is also relieved by a profuse discharge of urine, like those of Gelsem. and Verat. alb.

There may be tenderness of the scalp in the region of the coronal suture ; brushing or combing cause violent attacks of sneezing. The scalp in general is very sensitive to the touch, even of the hat. Carbo veg., China, and Merc. have the same.

In the disease of the eyes Silicea is often useful; in fistula lachrymalis Calc. carb., but when the bone is affected this remedy is the one. Cataract of the right eye has been cured by it in scrofulous subjects. The patient may have frequent attacks of momentary blindness. Ulcers of the cornea with fungus hæmatodes.

Hardness of hearing when the ears sometimes open with a loud report, and worse during the full moon, is curable by Silicea.

There is also hardness of hearing especially of the human voice, which is similar to the deafness of Phosphorus. There is also a deafness cured by Silicea which is partially relieved by blowing the nose (similar to Manganum). Hardness of hearing when yawning is accompanied by reports in the ears which improves the hearing.

There are ulcers on the vermillion border of the lips. The gums are painfully sensitive on taking cold water into the mouth.

The patient is annoyed by a sensation of a hair lying on the forepart of the tongue. Kali bi. has this sensation on the root of the tongue, and Arsen. in the throat. Silicea, like Pulsatilla, has toothache worse when eating warm food.

Water tastes badly (Aconite everything tastes badly except water) and vomits after drinking (Arsen. also).

The patients longs for red sandstone. Alumina has a similar depraved appetite for clay, dirt, etc. She has a repugnance to hot cooked food (Calc.). Aversion to meat; Ferrum has this also. These depraved tastes we will find frequently in chlorosis, abnormal conditions of pregnancy, and other complaints of women. She may be hungry, but cannot get down the food it is so nauseous.

We find certain forms of dyspepsia amenable to Silicea. After a meal a load as from a stone in the stomach, particularly after eating raw vegetables, and acidity in the mouth always after eating. Silicea cures some kinds of nausea. Nausea with violent palpitation of the heart; nausea after every exercise that raises the temperature of the body. Hungry, she cannot get down food, it is so nauseous. The patient may have frequent attacks of colic, which is relieved by the discharge of offensive flatus.

In the class of patients in which Silicea is indicated, we frequently find constipation, which is cured only by this remedy. Constipation; stools very hard and offensive. Constipation with constant ineffectual urging, and insufficient, very hard stools of small lumps (very much like that of Nux v.). The most characteristic constipation of this drug is that in which the stools are with difficulty forced to the verge of the anus, and then slip back again.

The piles cured by Silicea are disposed to suppurate easily.

Next to scrofulous children, the class most benefited by Silicea are scrofulous women, and the generative organs are disordered in many cases which require this remedy.

Leucorrhœa is nearly always present in psoric women. The forms indicating Sil. are the following: milky, watery, or brown leucorrhœal discharge, instead of the menses; leucorrhœa which passes while urinating; smarting, acrid, and corrosive leucorrhœa (Kali carb., Phos., and Sulph. resemble it); milky leucorrhœa in paroxysms preceded by cutting pain round the umbilicus. (Ammon mur. has colic in the same region before the leucorrhœal discharge.)

The symptoms of menstruation are characteristic and should be remembered. Before and during the menses the patient is costive; she has repeated paroxysms of icy coldness over the whole body at the appearance of the menses, and icy, cold feet during the menses.

During menses objects look blue. Menses are always accompanied by palpitation of the heart. The menstrual discharge smells strong. Patient has a discharge of a quantity of white water from the uterus instead of the menses. Menses occur during lactation. Instead of the menses she has an acrid leucorrhœa. She has discharges of blood between her regular periods. She may have an ulcer of the cervex which feels cold. She is troubled by a violent burning and soreness in the vulva with an eruption on the inner sides of the thighs. (Kali carb., Sulph.) After taking acids she has a smarting pain in the vulva. After coition there is a bruised feeling of the whole body, and during coition she is nauseated.

In the complaints of pregnancy we should always remember this medicine. If the patient cannot walk from a lameness or soreness in one or both feet from the instep, and all in front of and below it, and in the gastric complaints remember the nausea that I have already mentioned.

Nor would the armenitarium of the physician in the diseases following parturition be complete without Silicea.

In those diseases of the mammary glands, that to the scrofulous woman are sometimes more to be dreaded than childbirth itself, she has darting and burning pains in the left nipple.

The nipple ulcerates very easily and is very sore and tender. There are hard-edged fistulæ, ulcers remaining after mammary abscess, the discharge being thin and watery or thick and offensive.

The substance of the mamma seems to be discharged in the pus; one lobe after another seems to discharge into one common ulcer, often with pain, or there may be several orifices for each lobe.

When after-pains occur in the hips this is the remedy. If in the tibia, Carbo veg. is. If the lochia is such that every time the infant nurses pure blood flows from the vulva, give Silicea.

In diseases of scrofulous children Sil. plays an important part. The best time for curing the psoric taint is in antenatal life by meeting all the abnormalities of pregnancy. As I have shown; this remedy plays a great part in this.

But if this has been neglected, and the child, with all the effects of original sin (not syphilitic) is born into the world, again Silicea is one of the most useful remedies, standing with Sulph. and Calc. carb., and having a striking resemblance to the latter, and many times your success will depend on choosing *the right* one.

The child refuses the breast or vomits as soon as it nurses. In this Æthusa resembles it, but with the latter the milk comes up in clots. The child cries when kindly spoken to (Natrum mur., Calc., Phos. also), and will not tolerate friendly persuasion.

When teething it pulls at its gown.

In scrofulous children having worms with profuse salivation (here discriminate between Mercur. and Silic). The protruding gums seem blistered (Arsenic), and are very sensitive (Borax, Hepar, and Staph.), but in the case of children the appearance of the child must be our great reliance, and here Silicea has clear-cut indications, although with a striking resemblance to Calc. carb. Children with large bellies, weak ankles, much sweat about the head (just like Calc. carb.), and aggravation from uncovering it. But the sweat extends further down than with Calc. c., and is apt to smell offensive, with Calc. c. sour. But from personal experience I have found one distinguishing difference of these grand antipsoric remedies. Calc. carb. is indicated only in the fair, while Silicea in the dark, and according to Hering and Gross in the fair also. The fontanelles are large, and the head is larger in proportion than the rest of the body. (Calc. carb. also.)

In vermiculous subjects in whom Cina fails; but there is a difference in these children. When the child is sick at the change of the moon, although tolerably well at other times, Silicea is the remedy. In the scrofulous enlargement and suppuration of the glands, Silicea and Calc. carb. closely resemble each other. With Silicea there may be itching of the affected parts; with both there is usually no pain.

This is a remedy that is important in the treatment of tuberculosis, and is to be thought of at the same time as those other antipsorics, Calc. carb., Kali carb., Lycop., Phos., and Sulphur.

The cases which indicate Silicea have some of the following symptoms: Cough provoked by cold drinks (Calc. carb. and Lycop.), and relieved by warm drinks. Hasty eating and drinking brings on a cough. Patient hawks up very offensive balls of mucus. He expectorates pus, which when thrown into water falls to the bottom, and spreads like a heavy sediment. There is profuse mucus in the chest, which threatens to suffocate, and if raised is tough, stringy, and hard to detach (resembling Kali bichro.). Expectoration thick, yellow, and lumpy.

Silicea is specific in chest complaints of stone cutters.

It is in this and kindred diseases that the few febrile symptoms of this remedy have their field of action. He has great heat all night, with catching respiration. Coldness with ravenous hunger. Icy coldness of feet and legs. Profuse sweat every morning; with Arsenicum it begins as soon as he falls asleep, and soon ceases; with Phos. it begins as soon as he falls asleep, and continues as long as he sleeps. We also have a peculiar form of asthma, curable by this potent remedy, that which occurs only during thunder storms.

In the heart we have palpitation while sitting quietly so that the hand holding something trembles. Also palpitation during menses and during nausea.

Silicea is an important remedy in spinal diseases either of the cord or of the bones. These symptoms indicate Silicea: Tenderness in the region of the spine, so that riding or walking can only be performed with intolerable pain. The coccyx is painful as after a long ride.

In the hands we find burning in the tips of the fingers. The hands fall asleep at night. The tips of the fingers feel as if suppurating. In felons we must not forget this remedy. The indications are mostly constitutional.

This antipsoric is an important remedy in diseases of the joints, as hip-disease, and white swelling of the knee, but, like in the felon, we must look beyond the affected part.

The patient is troubled by profuse offensive sweat of the feet, and between the toes, the latter place being tender and sore, and if this sweat be suppressed, bad effects will sooner or later arise, for which Silicea still remains the remedy, together with Baryta carb. This offensiveness of the feet may exist even if there is no moisture present. Sometimes there are painful cramps in the sole of the right foot, and particularly in the great toe while walking.

Ingrowing toe-nails attended by this offensive sweat are cured by Silicea without the knife or forceps.

The Silicea patient is restless in the whole body after sitting a long time. Remember Rhus here. The whole side of his body on which he lies is sore as if ulcerated (with Arnica it is as if bruised), with constant chilliness on the slightest uncovering, with intolerable thirst, and frequent flashes of heat in the head. The whole body is painful as if beaten (like Arnica).

In epilepsy occurring at night (when falling asleep Lach.), or which returns at the change of the moon, Silicea is the remedy.

At night the Silicea patient is sleepless on account of unpleasant salivation, and he starts up during sleep as if in a fright. He starts up when falling asleep. Point to Arsen., Bell., and Bry. also.

When Silicea is required, the parts affected, particularly ulcers, are aggravated by washing. Symptoms are aggravated by cold and relieved by warmth (like Arsen.). The increase of the moon is always attended by aggravation.

In diseases of the glands, of the bones, and of the skin, this remedy does good work. In enlargement or suppuration of the glands, itching indicates Silic. These swellings are usually painless, like those of Calc. carb.

The differences between these remedies I have already mentioned.

Ulcerations which constantly increase in depth, becoming fistulous, and are usually surrounded by a hard border.

Silicea also may be indicated in chancres, which are sensitive, itch, and look like lard (Merc.). Small wounds in the skin heal with difficulty and easily suppurate (Hep.). Frequently ulcerations about the nails.

We have cachectic conditions, as from long lasting suppurations or cancer. Waxlike color of the skin, with coldness of the arms and legs objective and subjective.

The patient has a want of vital warmth even when taking exercise. Slow ossifications as manifested by crooked legs.

Some of the bones swollen, the angular shaft of the long bones become rounded, and the joints and abdomen too large.

In these conditions we must remember the points of agreement and difference between this remedy and Calc. carb.

It is a good antipsoric for those of negro blood, who are so often scrofulous.

The relation which this remedy holds to others must be remembered. It is often indicated after Calc. carb.; is complimentary to Thuja, and like that remedy is indicated in ailments following vaccination.

Graph., Fluor. ac., Hepar, and Lycop. follow it well. Silicea must not be given before or after Merc.

