# Correction

**Published:** 1887-09

**Authors:** 


CORRECTION.—June number, page 206, seventeenth line from bottom, for gown read *gums*.

